# Impact of conducting hand hygiene audits during the COVID-19 pandemic in an intensive care unit at a tertiary care centre in Kerala, India

**DOI:** 10.3205/dgkh000478

**Published:** 2024-04-30

**Authors:** Ardra Mohandas, Chithra Valsan, Sofia Joseph, Resmi Varghese, Jinsy Baby, Shibi Jacob, Nithya Sani, Shyby Babu

**Affiliations:** 1 Department of Microbiology, Jubilee Mission Medical College & Research Institute, Kerala, India; 2 Hospital Infection Control and Prevention unit, Jubilee Mission Medical College & Research Institute, Kerala, India

**Keywords:** hand hygiene, hand hygiene audit, adherence, healthcare workers, COVID-19

## Abstract

**Background:**

The COVID-19 pandemic era had witnessed an upsurge of healthcare-associated infections (HAI) in COVID intensive care units (ICUs), which can be reduced by following proper hand hygiene (HH) practice. Performing HH auditing in COVID ICUs and providing timely feedback to the stake holders is crucial to reduce HAIs.

**Methods:**

From July 2021 to December 2021, HH auditing was conducted in COVID ICUs. *Hand hygiene* (HH) complete adherence rate (HHCAR), HH partial adherence rate (HHPAR) and HH total adherence rate (HHTAR) were analysed. Profession-specific HHTAR and moment-specific HHTAR (for each WHO moment) were also calculated.

**Results:**

HHCAR, HHPAR and HHTAR were found to be 47%, 19% and 66%, respectively. There was a significant increase in the monthly HHTAR from 62.2% to 72.2% (p<.001). The profession-specific hand-hygiene adherence rate (HHAR) was found to be highest among nurses (67%), and the moment-specific HHAR of WHO-moments 2 (90%) and 3 (94.8%) had the highest HH adherence.

**Conclusions:**

Auditing HH and providing timely feedback significantly improved HH adherence. The greatest need now is to regularly conduct HH auditing in COVID locations of all healthcare facilities to reduce the HAI rate among the COVID-19 infected patients in ICUs.

## Zusammenfassung

**Hintergrund:**

Während der COVID-19-Pandemie kam es auf COVID-Intensivstationen zu einem Anstieg Healthcare-assoziierter Infektionen (HAI), die durch ordnungsgemäße Händehygiene (HH) reduziert werden können. Die Durchführung von Hygienekontrollen auf COVID-Intensivstationen und die rechtzeitige Rückmeldung an die Beteiligten sind entscheidend für die Reduzierung von HAI.

**Methode:**

Von Juli 2021 bis Dezember 2021 wurde auf COVID-Intensivstationen ein HH-Audit durchgeführt. Analysiert wurden die HH-Komplettadhärenzrate (HHCAR), die HH-Teiladhärenzrate (HHPAR) und die HH-Gesamtadhärenzrate (HHTAR). Die berufsspezifische HHTAR und die momentenspezifische HHTAR (für jedes WHO-Moment) wurden ebenfalls berechnet.

**Ergebnisse:**

Die HHCAR, HHPAR und HHTAR wurden mit 47%, 19% bzw. 66% ermittelt. Es gab einen signifikanten Anstieg der monatlichen HHTAR von 62,2% auf 72,2% (p<.001). Die berufsspezifische HH-Adhärenzrate (HHAR) war bei Krankenschwestern und -pflegern am höchsten (67%). Die zeitpunktspezifische HHAR der WHO-Momente 2 (90%) und 3 (94,8%) wiesen die höchste HH-Adhärenz auf.

**Schlussfolgerung:**

Die Auditierung der HH und die rechtzeitige Rückmeldung haben die Einhaltung der HH deutlich verbessert. Das Gebot der Stunde ist die regelmäßige Durchführung von HH-Audits an COVID-Standorten in allen Gesundheitseinrichtungen, um die HAI-Rate unter den COVID-19-infizierten Patienten auf Intensivstationen zu senken.

## Background

There is an acute surge of healthcare-associated infections (HAI) in COVID cares settings, especially intensive care units (ICU), most of which are due to multidrug resistant organisms (MDROs). The degree of illness of the patients and nature of the workload among healthcare workers (HCWs) are different in intensive care units (ICU) and non-ICU setups. The admitted patients stay for longer durations in ICU due to the severity of their disease, and they are more likely to be colonised with multidrug resistant organism (MDROs) [1]. When standard and transmission-based precautions are inappropriately/inadequately followed in any healthcare unit – including ICUs and non-ICU wards – transmission of HAI occurs at a higher rate. Hand hygiene (HH) is the single most effective way to decrease the rate of HAIs in COVID care locations. 

Although the awareness about HH among the general public and HCWs has increased during the COVID-19 pandemic [1], HH adherence continues to be low among the HCWs working inside COVID care settings. This can be attributed to increased work pressure, false beliefs that there is no need to do HH as all are COVID-positive patients, false beliefs that continuous use of gloves obviates the need for HH, and having a sense of discomfort due to continuous donning of personal protective equipment (PPE) [2].

To improve and maintain HH adherence in a sustainable way, performing HH auditing in COVID ICUs and providing timely feedback to the stake holders is crucial. Therefore, a prospective study was performed to determine the HH adherence rate among HCWs in the COVID ICU of our institution by conducting HH auditing, which can be used for quality improvement.

## Materials and methods

This was a prospective study conducted for 6 months (July 2021–December 2021) in a COVID ICU at Jubilee Mission Medical College, a tertiary care hospital located in Thrissur, Kerala, as a part of a prospective multicentric observational study with Jawaharlal Institute of Postgraduate Medical Education & Research (JIPMER) Pondicherry, as nodal centre. The study had ethical approval from the nodal center (JIP/IEC/2021/014 dated May 31, 2021) and from our Institutional Ethics Committee (IEC). The HH audit was performed to monitor the HH practice of HCWs posted in the COVID ICU. The HCWs – such as doctors (consultants and resident physicians), nurses, house-keeping staff and other staff (anaesthesia technicians, cardiovascular technicians) posted in the COVID ICU – were the study participants included in the audit.

The COVID ICU had 10 beds, with around 8–10 HCWs working at any given time point. The auditors selected to perform the HH audit were the infection control nurses (ICNs) of the Hospital Infection Control and Prevention (HICP) unit. The HH audit was conducted by direct observation method according to the WHO’s HH audit tool [3] and the data were collected electronically through an App (IBHAR HH audit App), provided by the nodal centre.

The HH event was marked as ‘completely followed’ when all 6 of the WHO steps of HH were performed [3], [4], [5] for the recommended duration (>20 seconds for handrub and >40 seconds for handwash). When ≥1 of the WHO’s HH steps were omitted and/or the duration was less than recommended, such HH events were marked as ‘partially followed’. The auditors also monitored and ensured the availability of consumables (e.g., handrubs, handwash, tissue papers) in the COVID ICU at all times. 

Immense efforts were taken to reduce all the possible bias expected to arise during the audit process and to ensure standardization and reliability of the audit. 

The HH audit was conducted for an observation period of 20 mins/d for a period of 6 months in the COVID ICU. The HH complete adherence rate (HHCAR), HH partial adherence rate (HHPAR) and HH total adherence rate (HHTAR, complete + partial) were calculated. Profession-specific HHTAR and moment-specific HHTAR (for each WHO moment) were also calculated [4], [6], [7]. The monthly HH audit report and the feedback were shared with the clinical team of the COVID ICU and also presented at the hospital infection control committee meeting.

The collected data was entered into Microsoft Excel and analysed using SPSS version 21 software (IBM-SPSS Inc, Armonk, NY). The month-wise HHTAR, profession-specific HHTAR and moment-specific HHTAR rates were reported as percentages, and the associations between the above-mentioned parameters were tested using the chi-square test and chi-square for trend. A P-value of <0.05 was considered statistically significant.

## Results

1,823 opportunities for conducting HH were recorded during the entire study period.

**Figure 1:**
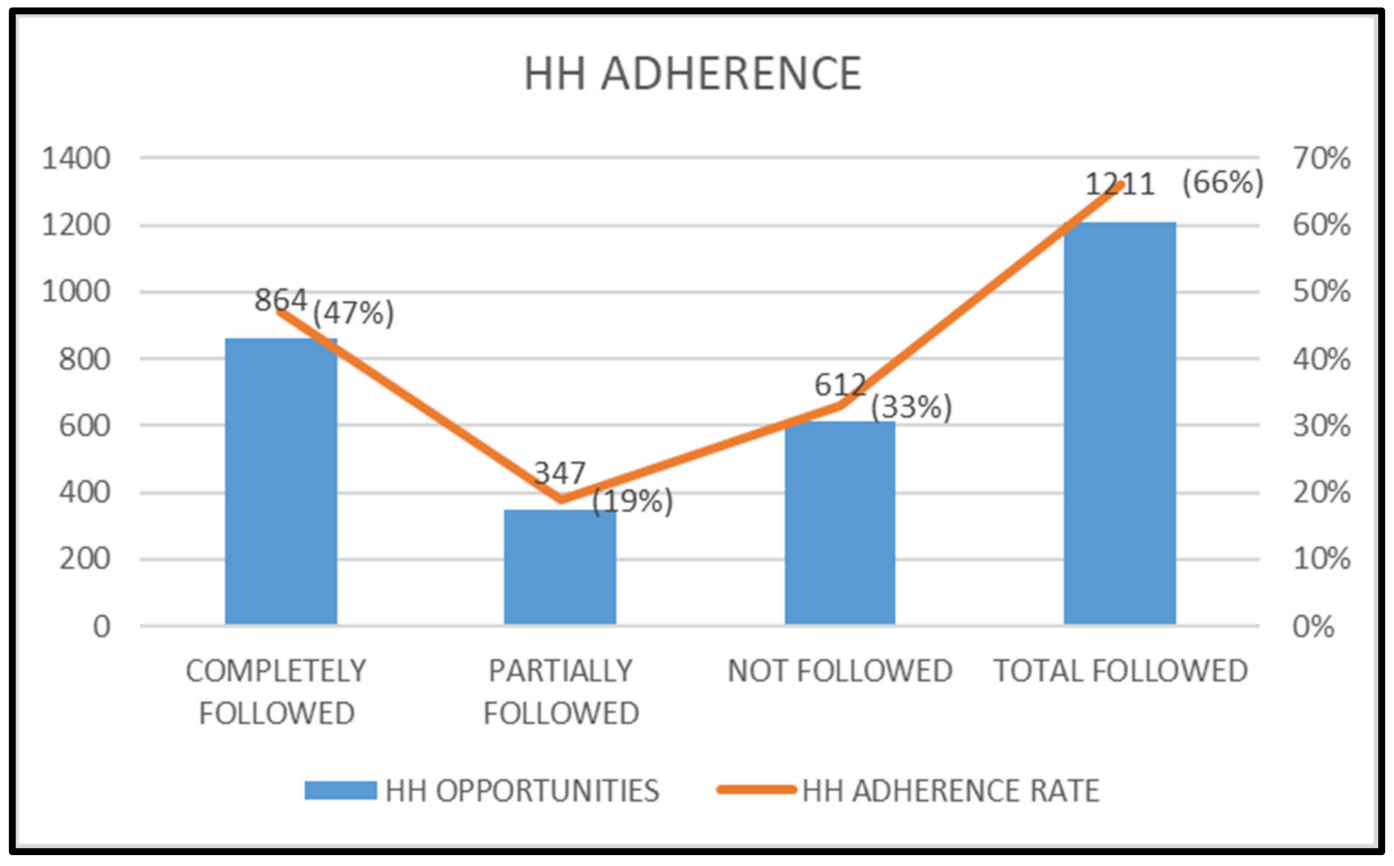
Adherence to hand hygiene

The HHTAR, HHCAR and HHPAR for the study period were found to be 66% (1211/1823), 47% (864/1823) and 19% (347/1823), respectively (Figure 1 (Fig. 1)). 

**Figure 2:**
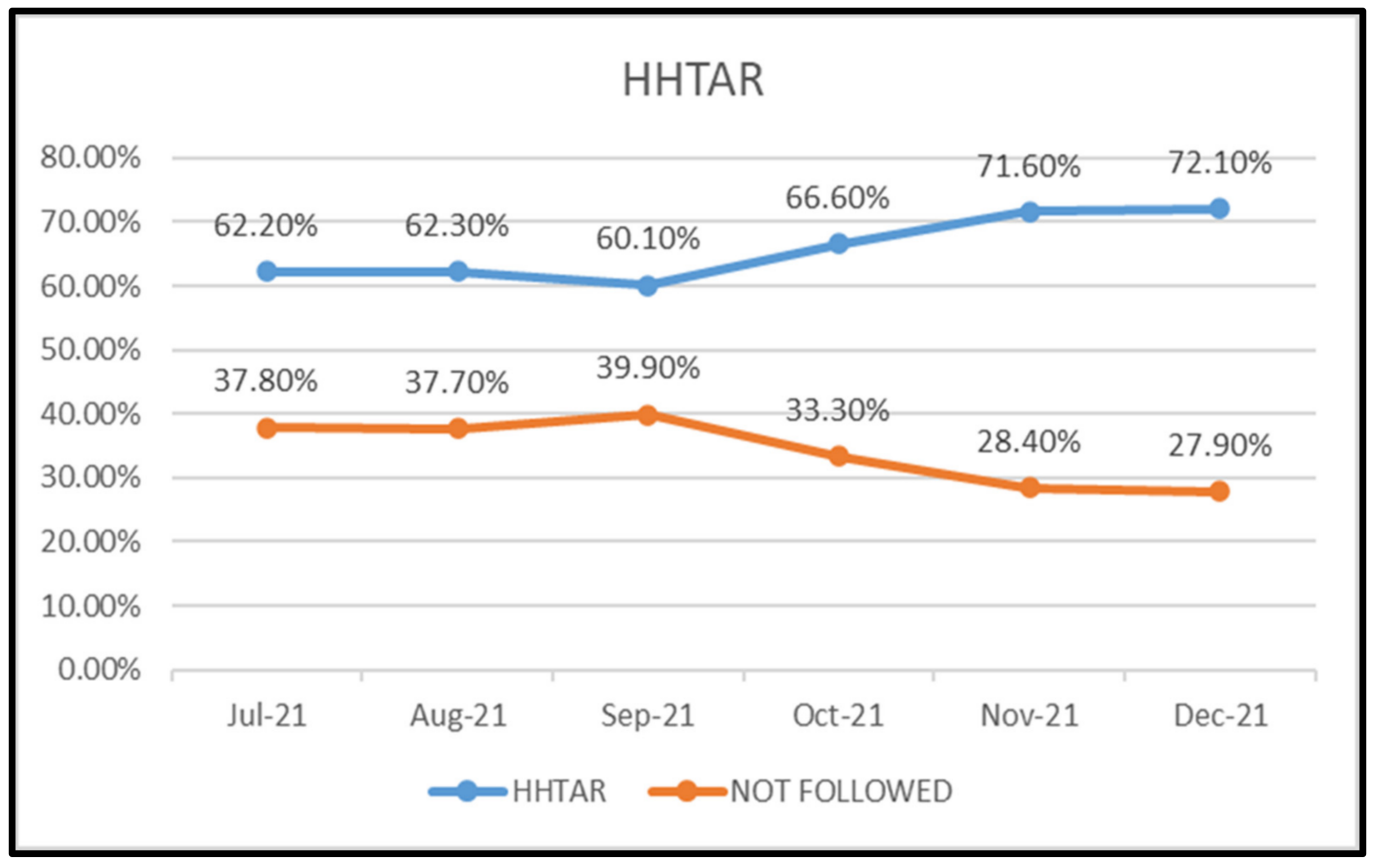
Total adherence rate of hand hygiene (HHTAR)

It was observed that monthly HHTAR progressively and significantly increased (p value <0.001) during the study period, from 62.2% to 72.1% ( Figure 2 (Fig. 2)).

**Figure 3:**
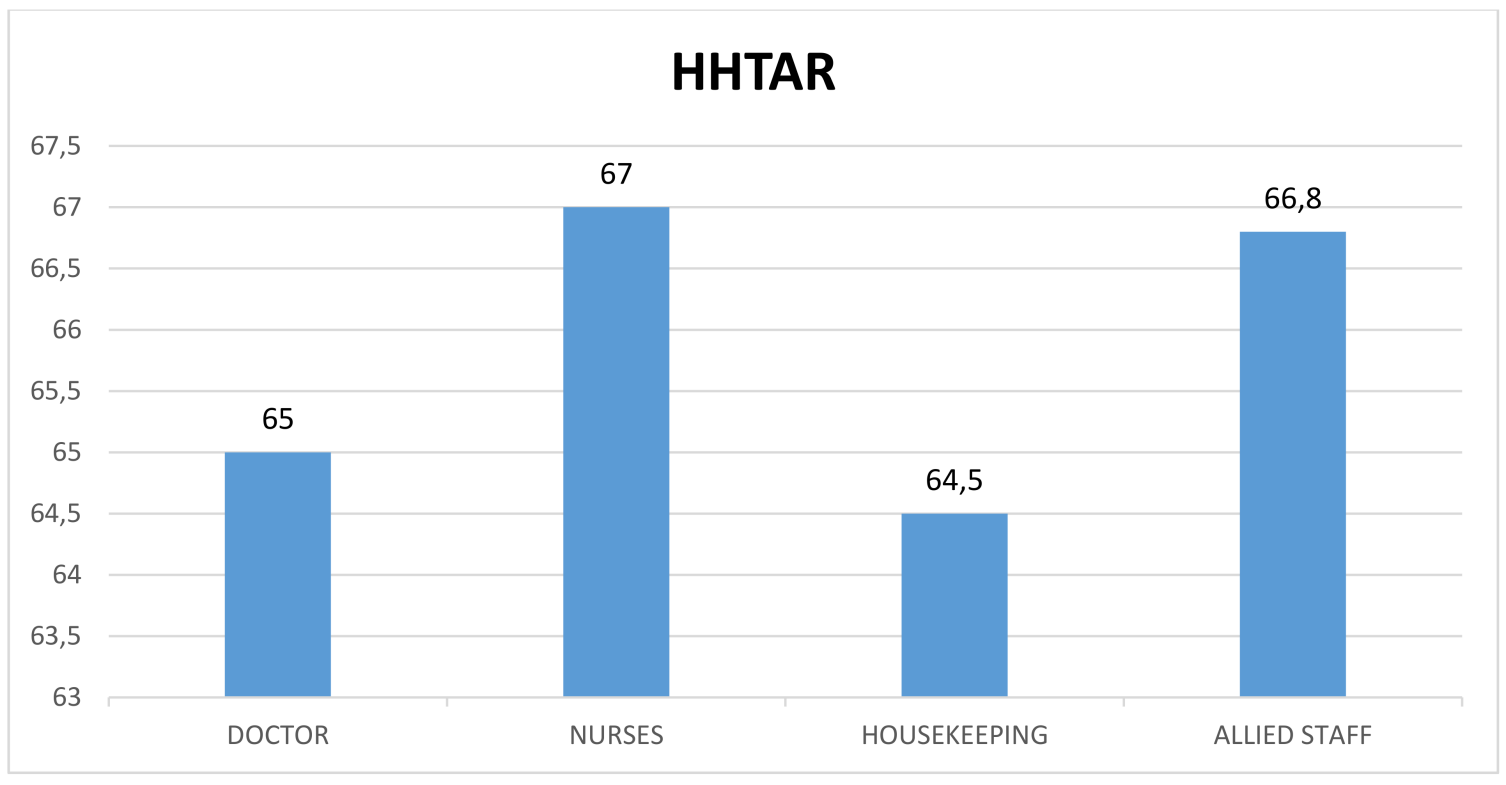
Profession-specific total adherence rate of hand hygiene (HHTAR) during the study period

The profession-specific HHTAR was found to be highest among nurses (67%) followed by allied staff, doctors and housekeeping staff (Figure 3 (Fig. 3)). 

**Figure 4:**
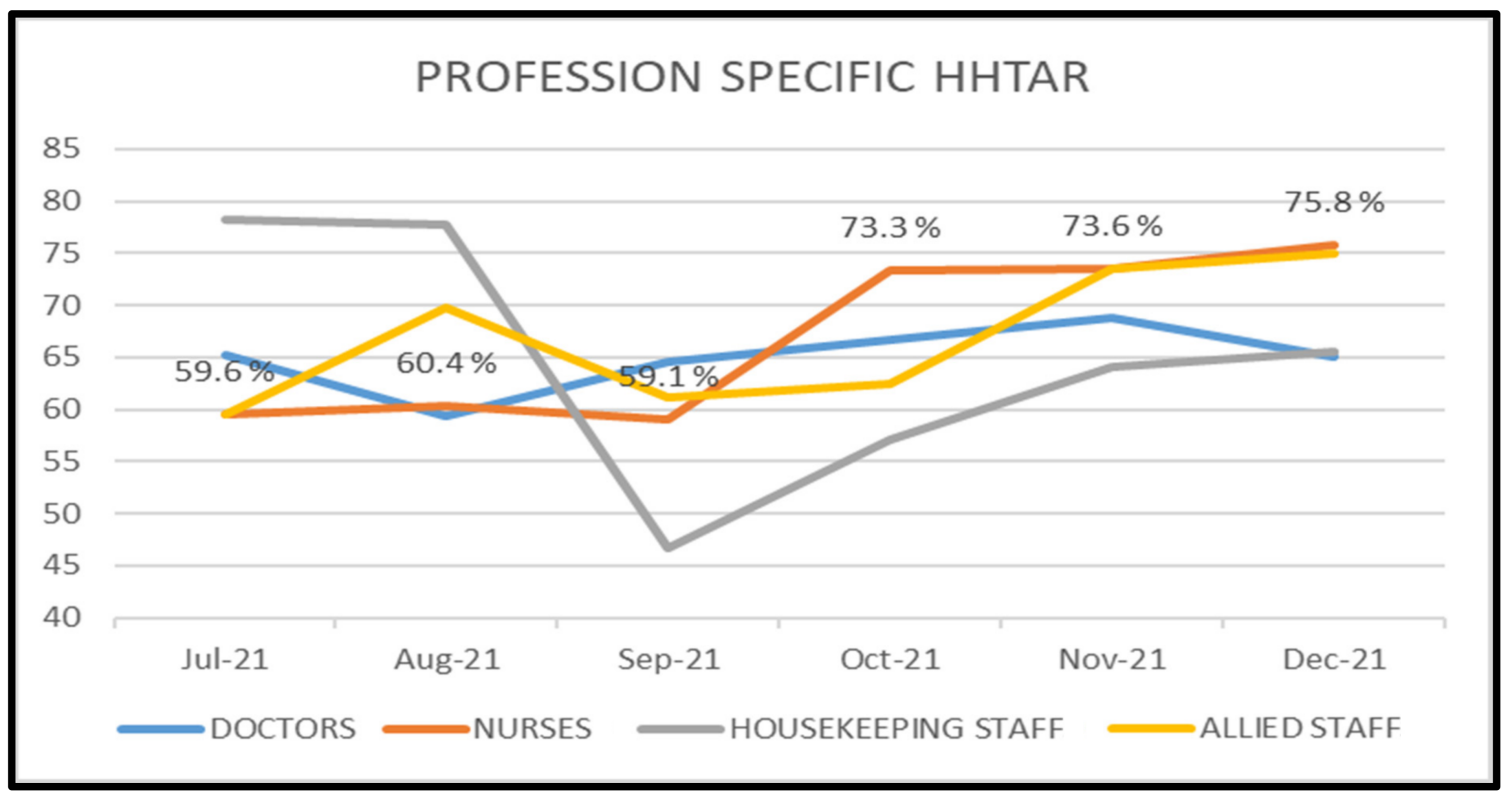
Monthly profession-specific total adherence rate of hand hygiene (HHTAR)

Whereas the monthly profession-specific HHTAR kept fluctuating among doctors, nurses, housekeeping and allied staffs, a statistically significant progressive increase was found only in nurses (Figure 4 (Fig. 4)).

**Figure 5:**
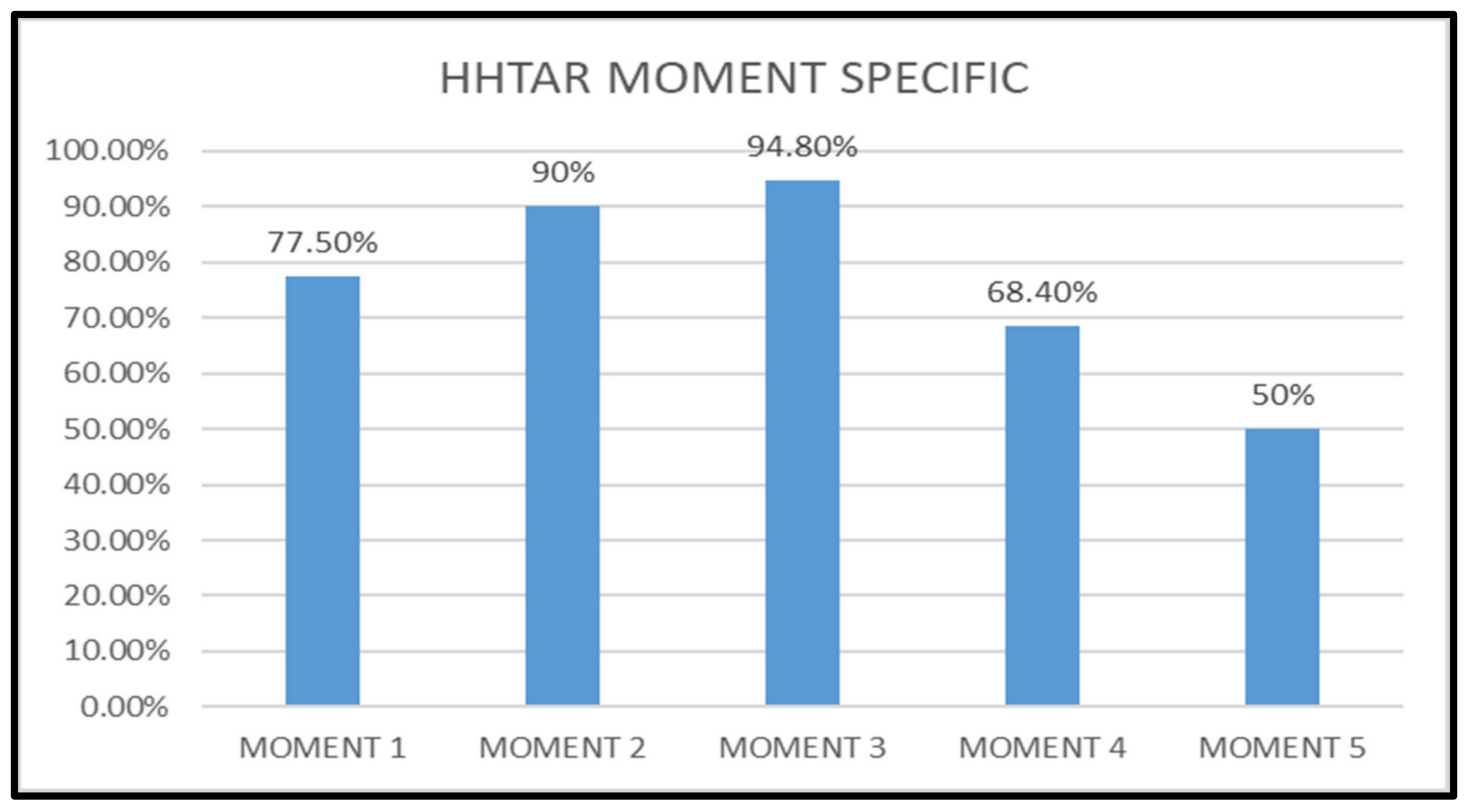
Total overall adherence rate of hand hygiene (HHTAR) for the 5 WHO-moments

In terms of moment-specific HH adherence, the WHO-moments 2 and 3 proved to have the highest HH adherence (90% and 94.8% respectively) (Figure 5 (Fig. 5)). 

**Figure 6:**
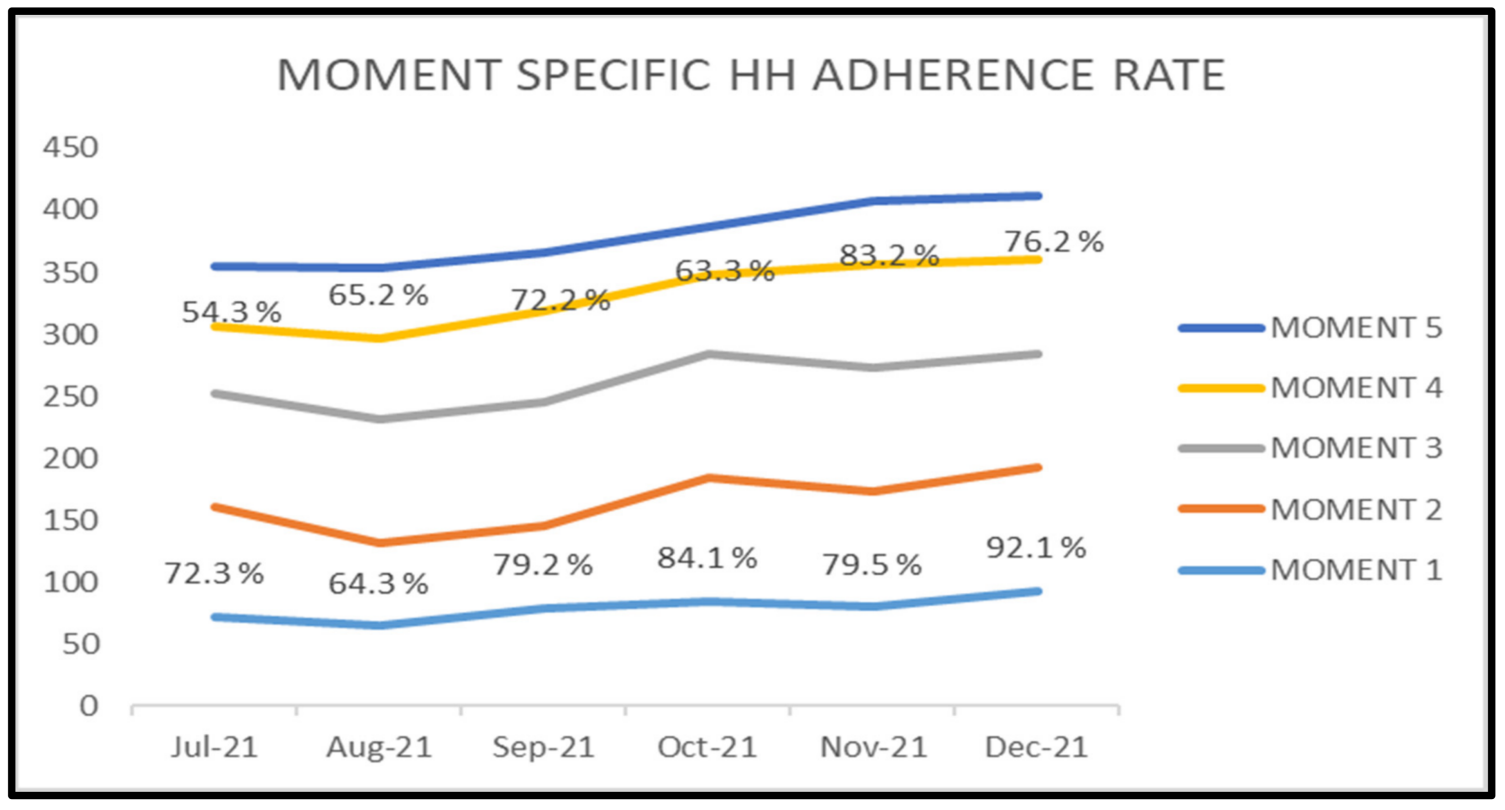
Trend of hand hygiene overall adherence rate (HHTAR) for each moment during the study period

The improvement in monthly HH adherence during the study period for moments 1 and 4 showed a statistically significant increase (p<0.001; Figure 6 (Fig. 6)).

## Discussion

This study was undertaken to monitor the HH practices of HCWs posted in the COVID ICU, with the objective of gradually improving HH adherence by providing timely feedback. HH nonadherence has contributed to the majority of MDRO- and fungi-related outbreaks in various ICUs during the COVID-19 pandemic [8]. The HH audit was conducted for a period of six months in a COVID ICU, during which a total of 1823 HH opportunities were observed.

A recent systematic review and meta-analysis showed an increase of HH adherence to 74% compared to pre-COVID pandemic studies [9]. The above adherence rates are comparable to those of our study (66%, 72.1%). We have made an effort to analyse HHTAR (complete and partial) in this study, although the WHO does not recommend monitoring partial adherence. Nevertheless, we attempted it in order to encourage the HCWs, in the hope that their partial HH adherence would become complete adherence in the subsequent audits. In the present study, the month-wise trend analysis showed that there was a significant improvement of HHTAR (62%–72%), HHCAR (37%–52%) and HHPAR (25%–20%) from July 2021 to Dec 2021. This signifies that providing feedback of HH performance to the HCWs on a daily basis and presenting HH audit reports in hospital infection-control committee meetings helps improve the adherence immensely.

A majority of studies [10] showed higher adherence in nurses, while very few studies showed higher adherence among doctors. The higher adherence among nurses could be explained by a relatively higher incidence of patient-care activities than other among other HCWs, which leads to establishing a habit of better HH practice. The month-wise trend analysis showed a progressive increase in the HHTAR towards the end of the study period, although it fluctuated between months among doctors, nurses (p<0.001) and ancillary staff. Similarly, Laskar et al. [11] documented a significant improvement in HHCAR among doctors (2.3%–50%) and nurses (3.6%–80%) following a multimodal intervention. 

The HCWs were continuously reminded to follow “My 5 Moments for HH” as emphasized by the WHO [4]. The “before” moments (moments 1 and 2) protect the patients from risk of microbial transmission from HCWs, whereas the “after” moments (moments 3, 4, and 5) prevent risk of microbial transmission from patients and their surroundings to HCWs. The “after”-moment HH adherence was better than the “before” moments; this may be due to fact that the HCWs tend to perform HH to protect themselves rather than the patients [12]. Similarly, increased adherence for the “before” moments was reported by Lohiya et al. [13]. In contrast, a few other studies demonstrated a HH adherence rate for the “after” moments [11], [14], [15], [16]. In our study, the HH adherence rates were found to be highest for moments 2 and 3 when compared to moments 1, 4 and 5. The explanation for this observation could be the increased concern of HCWs for HH when performing an aseptic procedure, compared to other moments which do not involve any invasive procedures. The month-wise trend analysis showed a progressive increase in moment-specific HHTAR, but a statistically significant improvement in HHTAR for moments 1 and 4. This implies the impact of HH auditing and feedback in shaping the HH practices of HCW, even lacking any aseptic techniques. A good HH adherence directly reflects in reduction in HAIs and eventually results in a more effective healthcare system.

## Conclusion

Regular HH auditing and timely feedback to the stakeholders have a significant positive impact on HH adherence in healthcare systems. A sustainable behavioural change despite increased work pressure is paramount to achieve a higher standard of HH adherence, as this simple infection-prevention measure plays a significant role in preventing cross-transmission of MDROs in healthcare settings.
